# AI Personal Assistants and Sustainability: Risks and Opportunities

**DOI:** 10.1021/acs.est.4c03300

**Published:** 2024-04-18

**Authors:** Matthias C. Rillig, Atoosa Kasirzadeh

**Affiliations:** 1 Freie Universität Berlin, Institute of Biology, 14195 Berlin, Germany; 2 Berlin-Brandenburg Institute of Advanced Biodiversity Research (BBIB), 14195 Berlin, Germany; 3 The University of Edinburgh, Edinburgh Futures Institute, Edinburgh EH3 9EF, U.K.; 4 Alan Turing Institute, London NW1 2DB, U.K.

**Keywords:** AI personal assistants, large language models, environmental education, environmental impact, digital divide, environmental literacy, artificial intelligence

In the near future, households and businesses may increasingly rely on advanced AI personal assistants (AIPAs) to manage their daily operations, with potentially transformative effects on energy use, carbon emissions, and sustainability. AIPAs are digital agents powered by generative AI, such as large language models (LLMs) like those that underpin ChatGPT, which can produce human-like text, image, and video.^1^ These AI-driven assistants interact with users through natural language interfaces, translating human requests into computer commands to manage various devices, tasks, and applications.^2^

Current AIPAs, such as Amazon’s Alexa, Microsoft’s Cortana, Google Assistant, or Apple’s Siri, are designed to assist with a relatively narrow set of tasks, and as such, they are far cry from Joi, the holographic and immensely capable AIPA featured in the movie “Blade Runner 2049”. However, as the rapid technological development in generative AI continues, future AIPAs may become capable of mastering highly complex tasks that involve content creation, device integration, communication, coaching, real-time information retrieval and processing, and organization. These advanced AIPAs could leverage the in-depth knowledge of personal preferences and habits, as well as immense local or regional databases, to optimize resource consumption and promote sustainable practices across households and businesses.

For example, an advanced AIPA could analyze a household’s energy consumption patterns, suggest personalized energy-saving measures, and automatically control appliances and systems to minimize waste. In an office setting, AIPAs could optimize lighting, heating, and cooling based on occupancy and weather conditions, while also encouraging employees to adopt eco-friendly habits, such as using public transportation or reducing paper consumption. At a larger scale, AIPAs could be integrated with smart city infrastructure to optimize resource allocation, reduce traffic congestion, and promote the adoption of renewable energy sources.

Effects of AIPAs, as almost always with new technology such as LLMs, can range from beneficial to deleterious.^3^ Here we anticipate the potential effects of this technology on the environment and sustainability, with the hope that the more positive effects will materialize, while raising awareness of the risks so that they can be mitigated.

## Potential Risks

The development of next-generation AIPAs involves having deep access to large amounts of users’ personal preferences and habits, offering highly personalized experiences. This access is facilitated on the basis of the data they have collected due to previous interactions and databases. This can have a number of consequences.

AIPAs may inadvertently reinforce users’ knowledge bubbles,^4^ potentially deprioritizing environmentally relevant topics if they are not already part of the user’s interests. The companies supplying these assistants may also have their own agendas, which could influence the information provided to users.

Like other generative AI systems,^5^ AIPAs are vulnerable to exploitation. The intimate nature of interactions with AIPAs could amplify the impact of such malicious activities. Malicious actors could use AIPAs to carry out targeted scams and fraud related to environmental issues. For instance, they might promote fake eco-friendly products, solicit donations for bogus environmental causes, or trick users into revealing personal information under the guise of sustainability initiatives. The trust users place in their AIPAs could make them more vulnerable to such deception. Perhaps most concerningly, bad actors could exploit AIPAs to manipulate user behavior in ways that undermine sustainability efforts. By subtly influencing the recommendations and suggestions provided by AIPAs, malicious actors could encourage users to make less eco-friendly choices, such as purchasing products with a higher environmental impact or engaging in energy-intensive activities. Over time, this manipulation could have a significant cumulative effect on sustainability.

Biases introduced during the training phase of AI models can affect outputs.^6,7^ These biases could, among other things, pertain to environmentally relevant information (e.g., pollution, sustainability, climate change, and biodiversity decline) and thus result in skewed output in the form of actions, text, advice, and communications. Increased time spent with technology may reduce meaningful in-nature experiences, leading to a deprioritization of environmental topics; this could have overall negative cumulative consequences for the environment and sustainability. Language barriers, digital literacy gaps, and socioeconomic disparities can limit the equitable availability of AIPAs, potentially reducing their impact on environmental conservation efforts.

Companies with poor environmental records may exploit AIPAs to engage in greenwashing, the practice of presenting a false or misleading image of environmental responsibility. By manipulating the information provided by AIPAs, these companies could attempt to mislead users about the sustainability of their products or services, undermining efforts to promote genuine eco-friendly alternatives.

In addition, there will also be direct environmental consequences, as is the case for other instances of generative AI use.^5−7^ The increasing use of advanced AIPAs contributes to significant energy consumption due to the computational power required for their operation.^6,8^ This exacerbates the carbon footprint associated with digital technologies, impacting the environment negatively. It is important to ensure renewable energies are being substituted in a timely manner. As technology evolves, older devices that support AIPAs become obsolete, leading to an increase in electronic waste. The disposal and management of this waste pose environmental hazards, including soil and water pollution. The production of devices capable of supporting AIPAs requires rare earth metals and other resources, the extraction of which can lead to habitat destruction, biodiversity loss, and other environmental impacts. The data centers that power AIPAs emit substantial amounts of carbon dioxide due to their energy-intensive operations. This contributes to climate change. The vast amounts of data generated and stored by AIPAs require more data centers, which consume significant energy resources and can lead to the degradation of natural habitats to accommodate these facilities.

There are concerns that might indirectly also affect environmental topics, but which we will not discuss at depth here. Such concerns include data privacy and security concerns, concerns related to reduced levels of human interaction,^9^ potential manipulative effects on cognition and behavior,^10^ and contribution of AIPAs to accumulative existential risks from advanced technologies.^11^

## Expected Positive Aspects

At the same time, there are excellent opportunities that AIPAs offer to achieve urban sustainability goals. We highlight three main points.

First, AIPAs could integrate vast amounts of data from various sources, including air quality sensors, traffic patterns, and weather forecasts. By understanding individual users’ habits and preferences, they can provide personalized environmental insights and recommendations tailored to their daily routines. For instance, an AI assistant could suggest eco-friendly routes for commuting, inform users about recycling, or help them switch to energy-saving modes when using appliances at home. This could translate to savings in energy use and carbon emissions.

Second, AIPAs could promote sustainable lifestyle habits. Maybe there is an “opt-in” or probably even better “opt-out” option for issues related to sustainability that could underpin the suggestions made by your AI personal assistant on a wide range of environmental topics. This would be potentially a great opportunity with environmental benefits. Might this also be a great opportunity for developers and researchers?

Finally, AIPAs can play a crucial role in educating the public about environmental issues and propel individuals to take action. They can provide access to personalized educational resources, offer tips to reduce environmental impact, and help connect people in relevant community groups and organizations. This can foster a sense of environmental stewardship and collective action in urban settings.

AIPAs, especially as they become integrated with other digital tools, local infrastructure, and information, hold huge promise to be a part of a sustainable transformation. However, this potential can be realized only when certain aspects during the development of these agents are enforced. Organizations designing these digital agents should prioritize algorithmic and model training transparency, and efforts should be made to prevent malicious actors from exploiting such systems. Even though this development is likely going to be driven by private companies, this would also be an opportunity for cities and local governments to become more actively involved, for example in partnerships. The time is ripe to infuse the development of such systems with the necessary components and safeguards to help maximize their environmental effectiveness in cities and beyond.
